# Editorial: Therapeutic Drug Monitoring (TDM): A Useful Tool for Pediatric Pharmacology Applied to Routine Clinical Practice

**DOI:** 10.3389/fphar.2022.931843

**Published:** 2022-05-24

**Authors:** Raffaele Simeoli, Thomas P. C. Dorlo, Lidwien M. Hanff, Alwin D. R. Huitema, Erwin Dreesen

**Affiliations:** ^1^ Division of Metabolic Diseases and Drug Biology, Bambino Gesù Children’s Hospital, IRCCS, Rome, Italy; ^2^ Department of Pharmacy and Pharmacology, Netherlands Cancer Institute, Amsterdam, Netherlands; ^3^ Department of Pharmacology, Princess Máxima Center for Pediatric Oncology, Utrecht, Netherlands; ^4^ Department of Clinical Pharmacy, University Medical Center Utrecht, Utrecht University, Utrecht, Netherlands; ^5^ Clinical Pharmacology and Pharmacotherapy Unit, Department of Pharmaceutical and Pharmacological Sciences, KU Leuven, Leuven, Belgium

**Keywords:** therapeutic drug monitoring, pediatrics, pharmacokinetic/pharmacodynamics, antimicrobials, personalized therapy

## 1 Background

For many therapeutic areas in modern medicine, precision medicine is the current treatment paradigm. Interestingly, or probably disappointingly, the concept of precision medicine often only encompasses selecting the right drug for the right patient. Subsequent selection of the right dose for the right patient is at least equally important and is only scarcely considered. Therapeutic Drug Monitoring (TDM) can be defined as assessing the adequacy of the drug plasma concentrations in relation to a target concentration or concentration window at a specific time in a dosing interval. This evaluation, following appropriate clinical interpretation and according to the drug pharmacokinetic/pharmacodynamic (PK/PD) properties, can guide dosing. However, finding the optimal dosing in order to guarantee a therapeutic exposure remains complicated. Sources of PK variability, including age, genetic heritage, and disease conditions, all influence the chances of achieving therapeutic outcomes. This aspect is particularly evident in children and neonates where physiological changes, associated with growth and maturation, dramatically influence drug PK properties. Similarly, pediatric patients subjected to medical procedures, including dialysis or extracorporeal membrane oxygenation (ECMO), may require special dose considerations since these procedures could significantly affect PK parameters. In pediatrics, drug-administration issues are more prominent than in adults. To adjust the dose and to secure intake in pediatrics, adult formulations may need to be crushed, dissolved, or extemporaneously prepared as liquid. In the absence of reliable data on these manipulations, TDM may contribute to safe and effective therapy in pediatric practice.

The utility of TDM is often underestimated or exclusively applied to molecules where monitoring of drug concentrations is mandatory due to safety concerns. However, TDM is less commonly used for optimization towards effective drug exposure. Although the multiple benefits of TDM are well known and recognized by clinicians, this knowledge seems to be only theoretical and its introduction into clinical practice remains far away from the application. TDM should rely on analytical methods such as high-performance liquid chromatography coupled to UV (HPLC-UV) or to mass spectrometry (LC-MS/MS) characterized by fast detection, and high accuracy and precision. These instruments are not always available for every drug and in every analytical laboratory, making TDM often unfeasible. Another issue that limits the application of TDM in pediatric patients is the invasiveness of blood sampling. The impact of blood sampling in children should not be underestimated. Even with professional psychological support, many children describe blood sampling as a dismal experience, and -naturally- TDM should therefore only be applied if clinically relevant. Low sampling volume limits in neonates and children make the bioanalytical procedures not always applicable, thereby limiting the use of TDM. To overcome this issue, several microsampling methods have been proposed including volumetric absorptive microsampling (VAMS) and dried blood spots (DBS) sampling. However, exhaustive analytical and clinical validation of these methods for conducting PK studies and TDM interventions is still rather limited.

## 2 Scope

The aim of this Research Topic was to show the utility of TDM applied to the routine clinical practice in pediatric patients. To this scope, we have collected reviews, research articles, case reports, and both pre-clinical and clinical study protocols that investigate the utility and the application of TDM in different pediatric settings and disease conditions. Moreover, an important contribution has been provided by population pharmacokinetic (popPK) studies that demonstrate how modelling and simulation approaches could provide important dosing guidance for drugs used *off-label* in neonates and children. These studies could be particularly useful to overcome the physiological and ethical issues connected to the realization of PK clinical studies on these subjects.

## 3 Overview of Contributions

### 3.1 Antimicrobial Therapy

Infections represent one of the main complications among hospitalized neonatal and pediatric patients, especially during long-standing periods in intensive care units (ICUs) where the presence of central catheters for parental nutrition and ventilators for respiratory support are often sources of bacterial colonization and require an appropriate shunt lock therapy to avoid systemic infections (Auriti et al., 2016; Ramasethu, 2017). Therefore, antimicrobial therapies are often introduced not only as therapeutic but also as prophylactic treatments. A further complication is represented by invasive procedures to which patients are often subjected. These include, for example, continuous renal replacement therapy (CRRT) and the ECMO. In these critical situations, drug administration (including antimicrobial agents) needs to consider the changes in PK parameters taking place in these patients. An increase in the distribution volume (Vd) or an augmented clearance of administered drugs can affect therapy effectiveness, exposing patients to a higher risk of therapeutic failures. An example of this important aspect has been described by Xu et al. These authors report two cases of critically ill pediatric patients with acute kidney injury requiring CRRT and receiving polymyxin B treatment due to carbapenem-resistant organism bloodstream infections. TDM of polymyxin B revealed an increased clearance of polymyxin B through CRRT that required supplanted dosing of the drug Xu et al. Similarly, a popPK analysis, aimed at describing primary PK/PD parameters of vancomycin and meropenem in pediatric patients undergoing ECMO, showed that the PK/PD target for vancomycin was achieved partially with conventional doses meanwhile higher dosing with extended infusion was needed in the case of meropenem Zylbersztajn et al. Even pathological conditions including hematological malignancies can affect PK properties of many administered drugs. An augmented renal clearance (ARC) is increasingly recognized in pediatric oncologic patients. In these immunocompromised subjects, broad-spectrum beta-lactams are commonly prescribed for empirical or selective treatment of bacterial infections. André et al. compared trough concentrations of meropenem and piperacillin in a cohort of unselected pediatric hematology-oncology patients stratified according to their estimated renal function as decreased, normal or with ARC, and to their neutrophil count. The results obtained by this retrospective evaluation showed that intermittent administration of meropenem and piperacillin often fails to ensure sufficient exposure in treated patients even at maximal recommended daily dosage, perhaps due to an increased drug clearance. Therefore, the authors suggest systematic TDM alongside assessment of renal function, as valid support for dosage adjustment in order to guarantee an appropriate antibiotic therapy in pediatric patients affected by malignancies André et al. These articles confirm that TDM may represent a valuable tool for assisting clinicians in optimizing antimicrobial exposure. A proof of concept in this direction has been provided by Gatti et al. The authors report their experience as clinical pharmacological advice (CPA) and conclude that a TDM-based real-time CPA may be particularly useful to optimize antimicrobial therapies in different challenging pediatric settings Gatti et al. The prospective EXPAT Kids study aims to evaluate PK/PD target attainment within the first 36 h after initiation of beta-lactam dosing in critically ill children. Schouwenburg et al. will also investigate the association between PK/PD target attainment and patient characteristics and clinical outcomes. Aiming to reduce the workload for medical staff, they will validate the use of residual material from heparinized astrup syringes for TDM. The results of the EXPAT Kids study are eagerly awaited and are expected to provide important new insights to improve clinical outcomes through better beta-lactam dosing.

### 3.2 Immunosuppressant Therapy

Hematopoietic stem cell transplantation (HSCT) remains the only curative treatment in several pediatric hematological pathologies. Although this therapeutic approach has significantly improved clinical outcomes of pediatric patients with malignant and non-malignant disorders, graft-versus-host disease (GVHD) is an important cause of morbidity and mortality in patients receiving HSCT (Ferrara et al., 2009). One of the most difficult challenges to further improving HSCT outcomes is reducing toxicity while maintaining efficacy of conditioning regimens that include combinations of chemo- and serotherapy prior to HSCT. In this context, the use of TDM represents a valid support for personalized dosing of various conditioning agents and could be particularly useful, especially in children for whom pathological conditions lead to a greater PK variability. Recently, van der Stoep et al. provided an overview of the possible relationships between PK parameters and clinical outcomes or toxicities for the most commonly used conditioning agents in pediatric HSCT van der Stoep et al. Brooks et al. developed a popPK model for tacrolimus intravenous continuous infusion in pediatric and young adult HCT patients Brooks et al. The model was implemented in a Bayesian dosing tool, thereby making it available at the point-of-care and allowing a clinical impact.

High-dose methotrexate (HD-MTX) is widely used in pediatric acute lymphoblastic leukemia (ALL) treatment regimens. A popPK model of HD-MTX has been developed by Gao et al. in Chinese pediatric patients with ALL with the noteworthy aim to establish a personalized dosage regimen. In particular, potential covariates such as age, body weight, and biochemical measurements (renal and liver function) on MTX PK disposition were investigated. The results obtained from this study revealed that body weight and serum creatinine levels (SCr) were significant covariates on the disposition of MTX. Therefore, this popPK model combined with an *a posteriori* Bayesian approach can be used to estimate individual PK parameters and optimize personalized MTX therapy for pediatric patients with ALL Gao et al.

### 3.3 Bioanalytical Techniques Applied to TDM

Different types of bioanalytical techniques are commonlyused in clinical laboratories for TDM. Historically, gas chromatography (GC), HPLC-UV, and LC-MS/MS are the most frequently used technologies to measure drug concentrations in different biological matrices. Immunoassays, including the enzyme-linked immunosorbent assay (ELISA), are also used for the quantification of several drugs. Usually, these assays are easily accessible for many laboratories and do not require highly specialized personnel. However, immunoassays are not available for all drugs monitored in clinical laboratories and their sensitivity, specificity, and accuracy toward the target compound is lower than GC, HPLC-UV, or LC-MS/MS. Therefore, the latter techniques are usually preferred to perform TDM although not always accessible to all laboratories. In a recent study, Xia et al. have analyzed plasma valproic acid (VPA) concentrations in 711 pediatric patients with epilepsy and compared a routine Enzyme Multiplied Immunoassay Technique (EMIT) with a validated in-house LC-ESI-MS/MS method on the same samples. Consistency between the two assays was evaluated using linear regression and Bland-Altman analysis. Results revealed that both methods were closely correlated although EMIT assay overestimated VPA levels in human plasma compared with LC-ESI-MS/MS method. Therefore, the authors conclude that switching from immunoassays to LC-based techniques for TDM of VPA deserves close attention and a therapeutic range of 35.0–75.0 μg/ml could be more feasible. However, further studies are required to evaluate the eligibility of this alternative range in clinical practice Xia et al.

Similarly, a novel peptide biosensor (P_ABL_)-ELISA assay has been developed and validated by Montecchini et al. to investigate ABL1 *in vitro* activity in four immortalized leukemic cell lines. The assay was further validated on blasts derived from an adult affected by chronic myeloid leukemia (*BCR-ABL1* positive) and a child affected by ALL (*BCR-ABL1* negative). Phosphorylation of P_ABL_ was inhibited after incubating *BCR-ABL1* positive cell lysates with imatinib, but not with ruxolitinib. In conclusion, the authors suggest that the P_ABL_-based ELISA assay provides a novel *in vitro* tool for screening both the aberrant ABL1 activity in *BCR-ABL1* like ALL leukemic cells and their potential response to tyrosine kinase (TK) inhibitors Montecchini et al.

### 3.4 Neonatal TDM

Neonatal pharmacology deserves particular attention due to the complexity of maturational and physiological changes that not only characterize neonates but also affect the response of these patients to pharmacological treatments. Although pre-terms are generally considered part of the neonatal population, they are physiologically and pharmacologically different from full-term neonates and, therefore, require specific considerations (Allegaert et al., 2007; Somani et al., 2016). For example, caffeine citrate is widely used to treat apnea of prematurity. However, maturation and genetic variation are responsible for high inter-individual variability in the clinical response to caffeine in preterm infants, making the optimal dose administered controversial. In a recent review of literature, Long et al. have evaluated the PK profile of caffeine in preterm infants alongside the safety and efficacy of different doses of caffeine, therapeutic concentration ranges, and the impact of genetic variability on caffeine therapy. Although safety and efficacy of standard-dose caffeine have been already assessed, evidence for the safety of higher administered doses is not yet explored. Therefore, the authors suggest the utility of TDM when dose optimization is required for preterm infants who lack clinical response to standard-dose caffeine. In fact, even polymorphisms in PD-related genes have a significant impact on the inter-individual variability in pharmacological response to caffeine, therefore an individualized therapy based on TDM data could be particularly useful during routine clinical practice Long et al.

## 4 Conclusion

In conclusion, TDM in pediatrics represents, similarly to adults, a useful tool that allows a more tailored drug administration and, therefore, the individualization of therapy to avoid risks of therapeutic failures or drug toxicity. The contributions to this issue of Frontiers in Pediatrics clearly demonstrate the value of TDM for tailoring the administration of various drugs in pediatric patients, mainly during antimicrobial and immunosuppressive therapies. However, various challenges remain, preventing a wide uptake in routine clinical care. Overcoming the bioanalytical issues and providing a software tool for model-informed dosing support may be key for TDM to grow into a user-friendly and reliable tool for improving treatments in children and neonates.
